# The parallel mediating effects of work rumination between organizational trust and work-family conflict among emergency department nurses

**DOI:** 10.1186/s12912-026-04560-9

**Published:** 2026-03-24

**Authors:** Xiaoli Yang, Yonglin Qi, Dongmei Zhang, Dajian Xia, Yunfang Xie

**Affiliations:** People’s Hospital of Dazu District, Chongqing, China

**Keywords:** Emergency department nurses, Work rumination, Organizational trust, Work-family conflict, Mediating effect

## Abstract

**Aim:**

To investigate the parallel mediating roles of affective rumination and problem-solving pondering (components of work rumination) in the relationship between organizational trust and work-family conflict among emergency department (ED) nurses.

**Background:**

Promoting the balance between work and family for ED nurses is a favorable guarantee for maintaining social order. Organizational trust, as a beneficial resource for alleviating work-family conflict, has a positive significance for achieving work-family balance. However, the mediating mechanisms of the two dimensions of work rumination—affective rumination and problem-solving pondering—in the relationship between organizational trust and work-family conflict remain unclear.

**Methods:**

A cross-sectional study design was adopted. From March to May 2025, a convenient sampling method was used to conduct a survey on 240 eligible emergency department nurses from 22 hospitals in Chongqing, who completed the General Information Questionnaire, Organizational Trust Scale, Work Rumination Scale and Work-Family Conflict Scale. Data analysis was performed via structural equation modeling.

**Results:**

The scores of organizational trust, affective rumination, problem-solving pondering and work-family conflict among emergency department nurses were (43.86 ± 10.52), (14.18 ± 4.24), (16.81 ± 4.07) and (48.30 ± 13.75) respectively. Nurses who were male, aged 30–40 years, married, had two or more children, and worked more than 40 h per week had higher scores of work-family conflict. Affective rumination and problem-solving pondering exerted parallel mediating effects in the relationship between organizational trust and work-family conflict, with the effect values being − 0.088 and − 0.131 respectively.

**Conclusion:**

Affective rumination and problem-solving pondering play parallel mediating roles in the association between organizational trust and work-family conflict among emergency department nurses. It is recommended that targeted intervention strategies be formulated for these two types of rumination to alleviate work-family conflict among emergency department nurses.

## Introduction

Work-family conflict refers to a state of role confusion experienced by individuals when confronting irreconcilable contradictions between work and family domains, and its essence lies in the conflict of individual roles [1]. The work in emergency departments is characterized by a fast pace, high pressure, and frequent violent incidents, making it more challenging for nurses to find a balance between work and family. Relevant studies have shown [2] that approximately 90% of emergency department nurses in China report moderate to high levels of work-family conflict. High levels of work-family conflict are significantly associated with impaired physical and mental health of medical staff, reduced quality of care, and hindered sustainable development of the healthcare system. Specifically, such conflict is not only correlated with health issues such as sleep disorders and increased suicide risk but also contributes to nursing shortages, decreased quality of nursing services, and serves as a significant positive predictor of employees’ turnover intention [3–6]. Organizational trust is defined as an individual’s recognition of and reliance on the competence and reliability of the organization, leaders, and colleagues [7]. High levels of organizational trust can create a favorable working environment, prompting individuals to exhibit more harmonious and tolerant behaviors, thereby enhancing job satisfaction and work well-being among nursing professionals [8, 9]. A relevant study indicated [10] that high organizational trust can mitigate the negative impact of work-family conflict on the job satisfaction of rural teachers. This may be attributed to the fact that when work-family conflict arises, high organizational trust enables the organization, leaders, and colleagues to serve as a stable “backing,” which helps stabilize work emotions and the working environment, thereby reducing the negative effects of conflict. The high-intensity and high-pressure nature of emergency work often prevents tasks from being fully completed during working hours, leading to non-working hours becoming an “extension of work time.” Stressful work events are repeatedly ruminated upon, resulting in a state of work rumination, which includes affective rumination and problem-solving pondering [11, 12]. Affective rumination focuses on the continuous internal consumption of negative emotional experiences at work, while problem-solving pondering is a creative process aimed at seeking solutions to work-related issues [13]. However, existing studies have mostly focused on describing the phenomenon of conflict or analyzing single influencing factors, with insufficient exploration of the interactive mechanisms between organizational and individual cognitive levels, especially the lack of systematic testing of potential mediating pathways.

The work-family boundary theory suggests that the daily role transition between work and family domains is a core challenge for individuals, and organizational support is a key boundary-crossing resource for achieving a smooth role transition [14]. For emergency department nurses, work pressure often persists psychologically even after leaving the workplace, making it difficult to disengage from the work role and resulting in boundary erosion. The conservation of resources theory is further introduced as a supplementary explanatory mechanism, which refers to the process by which individuals protect existing resources and develop new beneficial resources when facing difficulties [15]. As a critical psychological resource, the organization not only acts as a supporting condition to facilitate boundary management but may also be associated with nurses’ cognitive patterns outside of work (i.e., the two forms of work rumination: affective rumination as a depleting resource and problem-solving pondering as an enhancing resource), thereby exerting an indirect association with work-family conflict. This study aims to integrate the aforementioned theories to construct a relational path model starting from the resource of “organizational trust,” linking to the internal process of “work rumination,” and ultimately associating with “work-family conflict.” We hypothesize that: (1) Higher levels of organizational trust are associated with lower levels of work-family conflict. (2) Affective rumination and problem-solving pondering play mediating roles in the relationship between organizational trust and work-family conflict.

## Methods

### Study design and participants

This study adopted a cross-sectional survey design. Given the irregular working hours and high work intensity of emergency department nurses, strict random sampling was impracticable; thus, a convenient sampling method was used to ensure an adequate sample size within a limited time frame. Based on accessibility and willingness to cooperate, the research team contacted 22 hospitals with emergency departments in Chongqing through the in-hospital collaboration network. These 22 hospitals were general hospitals of different grades, distributed across the main urban areas and some districts/counties of Chongqing, aiming to reflect the overall status of emergency department nurses in Chongqing as much as possible. From March to May 2025, eligible in-service nurses were selected as research subjects from the emergency departments of the 22 hospitals in Chongqing. Inclusion criteria: (1) Having worked in the emergency department for at least 1 consecutive year; (2) Holding a valid clinical nursing practitioner qualification certificate; (3) Volunteering to participate in the questionnaire survey. Exclusion criteria: (1) Visiting advanced training personnel during the survey period; (2) Nurses in rotation at the emergency department; (3) Nurses who were not on duty for various reasons during the survey period, including advanced training outside the hospital, sick leave, personal leave and retirement. The sample size was estimated as 10 to 20 times the number of variables. The measurement items involved in the study model were mainly derived from the Organizational Trust Scale (13 items), Work Rumination Scale (10 items) and Work-Family Conflict Scale (18 items), with a total of 41 observed variables. Meanwhile, the model included 4 latent variables: organizational trust, affective rumination, problem-solving pondering and work-family conflict. Considering the number of observed variables and model complexity comprehensively, and reserving a 20% invalid questionnaire rate, the target sample size was determined to range from 240 to 480 cases. Due to time and resource constraints during the actual questionnaire collection, a total of 240 valid samples were finally included, which met the sample estimation requirements and was comparable to the sample size of similar cross-sectional studies.

### Measures

#### General information questionnaire

Based on a literature review, the General Information Questionnaire was developed collaboratively by the research team, which included the following variables: gender, age, educational background, marital status, childbearing status, hospital grade, employment type, years of emergency nursing experience, professional title, administrative position, weekly working hours, monthly night shift frequency, and average monthly income.

#### Organizational trust scale (OTS)

The OTS was compiled by Chen et al. [16], comprising 13 items with three dimensions: hospital trust (Items 1–5), leadership trust (Items 6–9), and colleague trust (Items 10–13). The scale used a 5-point Likert scoring system, with scores ranging from 1 (strongly disagree) to 5 (strongly agree), where a higher total score indicated a higher level of organizational trust. The Cronbach’s α coefficient of the OTS was 0.936 when applied to nurses in Grade A tertiary hospitals [17], and it was 0.932 in this study, indicating acceptable internal consistency for the current sample. Confirmatory Factor Analysis (CFA) was conducted, and the results showed a good fit of the three-factor model (*χ*^*2*^*/df* = 1.283, Root Mean Square Error of Approximation [RMSEA] = 0.038, Comparative Fit Index [CFI] = 0.965, Tucker-Lewis Index [TLI] = 0.958, Standardized Root Mean Square Residual [SRMR] = 0.042). All items had standardized factor loadings ranging from 0.62 to 0.84 (*p* < 0.001). The overall Composite Reliability (CR) of the scale was 0.935, and the Average Variance Extracted (AVE) was 0.61; each dimension had a CR ≥ 0.86 and an AVE ≥ 0.53, demonstrating good reliability and validity of the OTS in the current sample.

#### Work rumination scale (WRS)

The WRS was developed by the Cropley team [18], including 10 items divided into two dimensions: affective rumination (Items 1–5) and problem-solving pondering (Items 6–10). A 5-point Likert scale was adopted, with scores from 1 (never) to 5 (always), and a higher score reflected a higher level of work rumination. For full-time employees across various industries, the Cronbach’s α coefficients of the affective rumination and problem-solving pondering subscales were 0.91 and 0.88, respectively [19]; in this study, the coefficients were 0.865 and 0.842, respectively, indicating acceptable internal consistency of the two subscales. CFA results revealed a good fit of the two-factor model (*χ*^*2*^*/df* = 1.315, RMSEA = 0.040, CFI = 0.957, TLI = 0.949, SRMR = 0.045), with all standardized factor loadings of items ranging from 0.60 to 0.82 (*p* < 0.001). For the affective rumination dimension, CR = 0.868 and AVE = 0.56; for the problem-solving pondering dimension, CR = 0.845 and AVE = 0.54. The overall CR and AVE of the WRS were 0.902 and 0.55, respectively, confirming good reliability and validity of the scale.

#### Work-family conflict scale (WFCS)

The WFCS was translated and revised by Zhang et al. [20], consisting of 18 items with three dimensions: time-based conflict (Items 1–6), strain-based conflict (Items 7–12), and behavior-based conflict (Items 13–18). The scale used a 5-point Likert scoring method, with scores from 1 (strongly disagree) to 5 (strongly agree), where a higher score indicated a stronger level of work-family conflict. The Cronbach’s α coefficient of the WFCS was 0.937 when used to investigate frontline night-shift nurses [21], and it was 0.936 in this study, suggesting acceptable internal consistency for the current sample. CFA results showed a good fit of the three-factor model (*χ*^*2*^*/df* = 1.402, RMSEA = 0.043, CFI = 0.948, TLI = 0.940, SRMR = 0.048). All items had standardized factor loadings between 0.61 and 0.83 (*p* < 0.001). Each dimension had a CR ≥ 0.87 and an AVE ≥ 0.52; the overall CR and AVE of the WFCS were 0.938 and 0.58, respectively, which met the research requirements for reliability and validity.

### Data collection and processing

#### Data collection

After obtaining the informed consent of the affiliated institutions and departments of the research subjects, an online questionnaire survey was conducted via the Wenjuanxing platform for data collection. The researchers sent the official questionnaire link to the head nurses of the emergency departments in each hospital, and the principal investigator provided explanations on questionnaire-related issues to them, inviting them to act as quality supervisors. After clarifying the inclusion and exclusion criteria, the head nurses organized eligible staff in their departments to complete the online questionnaire uniformly in the departmental study room. Participants decided whether to participate after reviewing the research purpose and content, and could withdraw from the survey at any time. Before the start of the survey, the devices were set to do-not-disturb mode, and a unified instruction was displayed on the first page for respondents, clearly stating the research purpose, confidentiality principles, voluntary participation and anonymity of the survey. Participants were required to check “Agree” on the electronic informed consent page to access the official questionnaire. The questionnaire was set to allow only one response per IP address, and all items were mandatory to prevent data duplication and loss. For participants unfamiliar with online operation, the head nurses provided on-site assistance (e.g., device operation or brief guidance) to ensure the sample was not limited to technically proficient individuals. All data were anonymized after export, and the data files were encrypted and stored on a dedicated computer, with access restricted exclusively to members of the research team.

#### Data validation and cleaning

To ensure data quality, the following data validation and cleaning steps were implemented in this study. First, invalid questionnaires were excluded based on the following criteria: patterned responses (selecting the same option for more than 10 consecutive items or showing an obvious response pattern), excessively short response time (less than 3 min to complete the questionnaire), contradictory key information, extreme responses (selecting the extreme option of 1 or 5 for all scale items), and logical errors (e.g., age outside the range of 18–55 years, working experience of less than 1 year). Second, reverse-scored item processing was conducted: none of the three scales used in this study contained reverse-scored items, and all items were positively worded, with response options scored from 1 to 5 in ascending order (e.g., from “strongly disagree” to “strongly agree”). Therefore, no reverse scoring conversion was required in this study. Finally, for the cleaned valid data, the internal consistency reliability (Cronbach’s α coefficient) of each scale was calculated as an auxiliary verification of data quality. A total of 253 questionnaires were collected in this survey; 13 invalid questionnaires were excluded according to the above criteria (5 with patterned responses, 4 with excessively short response time, 2 with contradictory key information, and 2 with extreme responses), resulting in 240 valid questionnaires with an effective recovery rate of 94.86%. For the 240 valid datasets included in the analysis, no influential univariate outliers were identified via box plot inspection, and no significant multivariate outliers were detected by calculating the Mahalanobis distance (*p*<0.001).

### Statistical methods

Data analysis was performed using the Statistical Package for the Social Sciences (SPSS) 26.0. Continuous variables were described as mean ± standard deviation, and categorical variables as frequencies and constituent ratios. The normality of variables was tested by calculating skewness and kurtosis coefficients; the absolute values of skewness for all variables ranged from 0.01 to 1.52, and the absolute values of kurtosis from 0.03 to 2.87. Multicollinearity was examined by calculating the Variance Inflation Factor (VIF), with VIF values ranging from 1.2 to 3.8, which were far below the critical value of 10. Common method bias was assessed via Harman’s single-factor test, and the explained variance of the first factor was 28.7%, lower than the critical value of 40%. Pearson correlation analysis was used to examine the associations among variables. In Analysis of Moment Structures (AMOS) 26.0, CFA was first conducted for each scale to test the fit of the measurement model, with *χ*^*2*^*/df*, Root Mean Square Error of Approximation (RMSEA), Comparative Fit Index (CFI), Tucker-Lewis Index (TLI), and Standardized Root Mean Square Residual (SRMR) as evaluation indices. Meanwhile, composite reliability (CR) and average variance extracted (AVE) were calculated to test convergent validity. A structural equation model (SEM) was then constructed, with organizational trust as the independent variable, affective rumination and problem-solving pondering as parallel mediating variables, and work-family conflict as the dependent variable. The maximum likelihood method was used for parameter estimation, and model fit was evaluated using *χ*^*2*^*/df*, RMSEA, CFI and TLI. The bias-corrected Bootstrap method (with 5000 resamplings) was applied to test the mediating effects, with a 95% confidence interval (CI) set. The test level was set at α = 0.05.

## Results

### Participant characteristics

A total of 240 emergency department nurses were enrolled in this study, with the demographic characteristics as follows: 56 males (23.33%) and 184 females (76.67%); 84 nurses aged under 30 years (35%), 123 aged 30–40 years (51.25%), and 33 aged over 40 years (13.75%); 75 unmarried (31.25%), 144 married (60%), and 21 divorced (8.75%); 89 childless (37.08%), 82 with one child (34.17%), and 69 with two or more children (28.75%); 46 with college diploma (19.17%), 189 with bachelor’s degree (78.75%), and 5 with master’s degree or above (2.08%); 164 with contract employment (68.33%), 24 with personnel agency (10%), and 52 with career establishment (21.67%); 137 with primary professional title (57.08%), 97 with intermediate title (40.42%), and 6 with senior title (2.5%); 207 without administrative position (86.25%), 22 as charge nurses (9.17%), and 11 as head nurses (4.58%); 84 with 1–5 years of emergency nursing experience (35%), 49 with 6–10 years (20.42%), and 107 with more than 10 years (44.58%); 45 with 0 night shifts per month (18.75%), 70 with 1–5 night shifts (29.17%), 84 with 6–10 night shifts (35%), and 41 with more than 10 night shifts (17.08%); 72 working ≤ 40 h per week (30%) and 168 working more than 40 h per week (70%); 22 with an average monthly income under 5,000 RMB (9.17%), 197 with 5,000–10,000 RMB (82.08%), and 21 with more than 10,000 RMB (8.75%); 194 from Grade A tertiary hospitals (80.83%), 26 from Grade B tertiary hospitals (10.83%), 2 from Grade C tertiary hospitals (0.83%), and 18 from Grade A secondary hospitals (7.5%).

### Descriptive statistics

Table 1 shows the current status of organizational trust, work rumination, and work-family conflict among emergency department nurses. The organizational trust score was (43.86 ± 10.52), the affective rumination score was (14.18 ± 4.24), the problem-solving pondering score was (16.80 ± 4.07), and the work-family conflict score was (48.30 ± 13.75).

**Table 1:** Scores of organizational trust, work rumination, and work-family conflict among ED nurses (*n* = 240)

Variable	Items	Item Score (Mean ± SD)	Total Score (Mean ± SD)
Organizational Trust	13	3.37 ± 0.81	43.86 ± 10.52
Hospital Trust	5	3.35 ± 0.91	16.73 ± 4.55
Leadership Trust	4	3.43 ± 0.94	13.72 ± 3.76
Colleague Trust	4	3.35 ± 0.98	13.42 ± 3.91
Work Rumination	10	3.10 ± 0.47	30.99 ± 4.75
Affective Rumination	5	2.83 ± 0.85	14.18 ± 4.24
Problem-Solving Pondering	5	3.36 ± 0.81	16.80 ± 4.07
Work-Family Conflict	18	2.68 ± 0.76	48.30 ± 13.75
Time-Based Conflict	6	2.68 ± 0.87	16.10 ± 5.22
Strain-Based Conflict	6	2.72 ± 0.88	16.33 ± 5.29
Behavior-Based Conflict	6	2.65 ± 0.89	15.88 ± 5.37

### Comparison of work-family conflict scores among emergency department nurses with different characteristics

Table 2 shows male, aged 30–40, married, having two or more children, and working over 40 h per week were significant factors influencing work-family conflict.

**Table 2:** Comparison of work-family conflict scores by characteristics

Characteristic	Group	WFC Score (Mean ± SD)	t/F	*p*
Gender	Male(*n =* 56)	54.63 ± 13.14	4.06	0.000^**^
	Female(*n =* 184)	46.37 ± 13.38		
Age (years)	< 30(*n =* 84)	46.76 ± 12.88	3.652	0.027^*^
	30–40(*n* = 123)	50.46 ± 13.96		
	> 40(*n =* 33)	44.12 ± 13.99		
Marital Status	Unmarried(*n =* 75)	43.55 ± 13.54	8.305	0.000^**^
	Married(*n =* 144)	51.12 ± 12.64		
	Divorced(*n =* 21)	45.90 ± 17.04		
Children	None(*n =* 89)	45.34 ± 13.78	3.492	0.032^*^
	One Child(*n =* 82)	49.49 ± 14.27		
	Two or More(*n =* 69)	50.70 ± 12.54		
Weekly Work Hours	≤ 40 h(*n =* 72)	43.78 ± 13.75	11.602	0.001^**^
	> 40 h(*n =* 168)	50.23 ± 13.32		

* *p*<0.05;** *p*<0.01

### Variable correlation analysis

Table 3 shows variable correlation analysis in the hypothesized model. The results show that Organizational trust is negatively correlated with work-family conflict and affective rumination respectively (*r* = -0.582, *p* < 0.01; *r* = -0.337, *p* < 0.01), and a positive correlation with problem-solving pondering (*r* = 0.437, *p* < 0.01). Affective rumination was positively correlated with work-family conflict (*r* = 0.442, *p* < 0.01), while problem-solving pondering was negatively correlated with work-family conflict (*r* = -0.488, *p* < 0.01).

**Table 3:** Correlation among variables (*n* = 240)

Variable	Affective Rumination	Problem-Solving Pondering	Organizational Trust	Work-Family Conflict
Affective Rumination	1.000			
Problem-Solving Pondering	-0.348^**^	1.000		
Organizational Trust	-0.337^**^	0.437^**^	1.000	
Work-Family Conflict	0.442^**^	-0.488^**^	-0.582^**^	1.000

^**^p<0.01

### Parallel mediating effects of work rumination between organizational trust and work-family conflict among emergency department nurses

**Fig. 1:**
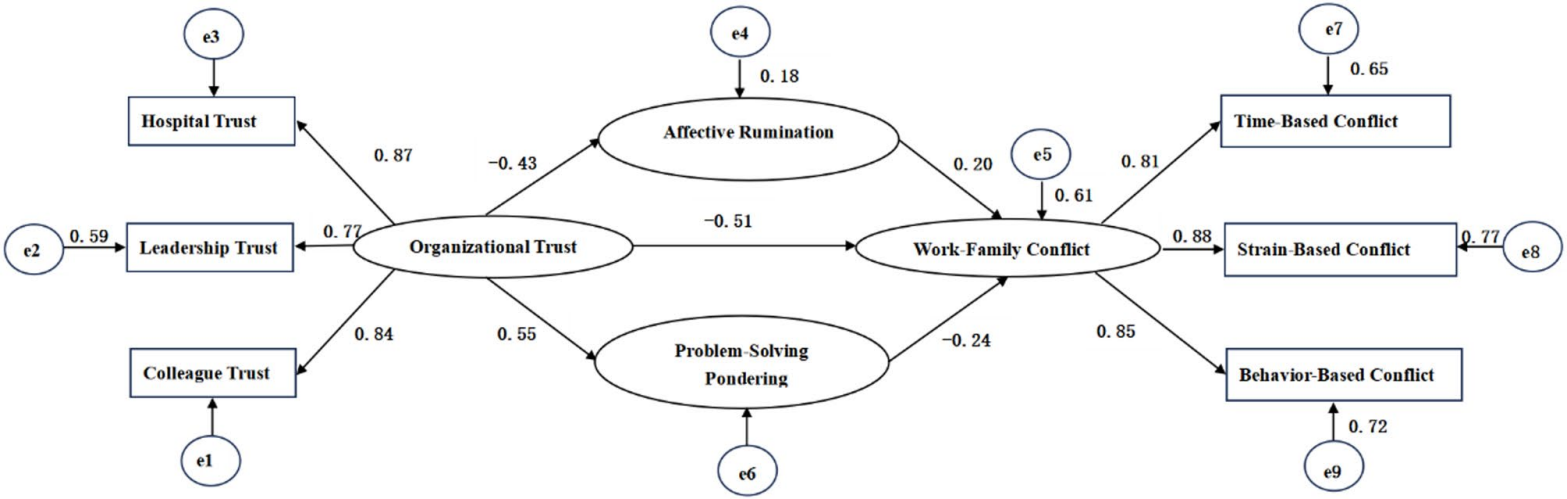
SEM of organizational trust, work rumination, and work-family conflict

With organizational trust as the predictor variable, work-family conflict as the outcome variable, and the two dimensions of work rumination (affective rumination and problem-solving pondering) as mediating variables, a hypothetical relationship was established, and a structural equation model(SEM)was constructed using Analysis of Moment Structures (AMOS) 26.0, as shown in Fig. 1. Modification indices were not used to modify the model. The model fit test showed that χ^2^/df = 1.392, Root Mean Square Error of Approximation (RMSEA) = 0.041, Comparative Fit Index (CFI) = 0.949, Incremental Fit Index (IFI) = 0.950, and Tucker-Lewis Index (TLI) = 0.941; all indices met the fit criteria, indicating a good model fit. Organizational trust was negatively associated with affective rumination (β=-0.428, *P* < 0.001) and work-family conflict (β=-0.515, *P* < 0.001), and positively associated with problem-solving pondering (β = 0.545, *P* < 0.001). Affective rumination was positively associated with work-family conflict (β = 0.204, *P* = 0.003), while problem-solving pondering was negatively associated with work-family conflict (β=-0.239, *P* = 0.002). The mediating effects were tested via the Bootstrap method with 5000 random resamplings of the original data, and the 95% confidence interval (CI) was estimated. The direct effect of organizational trust on work-family conflict was − 0.515. The mediating effect of organizational trust on work-family conflict through affective rumination was − 0.088, accounting for 14.6% of the total effect; the mediating effect of organizational trust on work-family conflict through problem-solving pondering was − 0.131, accounting for 20.3% of the total effect. Details are presented in Table 4.

**Table 4:** Analysis of the mediating effect of work ruminations between organizational trust and work-family conflict among emergency department nurses

Path	Effect Type	Effect Value	SE	Lower Limit	Upper Limit	*p*	Effect Proportion
Overall Organizational Trust → Work-Family Conflict	Direct Effect	-0.515	0.082	-0.666	-0.343	< 0.001	70.16%
Overall Organizational Trust →Affective Rumination → Work-Family Conflict	Mediating Effect 1	-0.088	0.032	-0.161	-0.034	0.003	11.99%
Overall Organizational Trust → Problem-Solving Pondering → Work-Family Conflict	Mediating Effect 2	-0.131	0.045	-0.231	-0.050	0.002	17.85%
	Total Mediating Effect	-0.219	0.051	-0.328	-0.130	< 0.001	29.84%
	Total Effect	-0.734	0.051	-0.834	-0.611	< 0.001	100%

## Discussion

### Current status and influencing factors of work-family conflict among emergency department nurses

This study found that the overall level of work-family conflict among emergency department nurses was moderately high, with a score of (48.30 ± 13.75). This result was close to that of some studies conducted after the COVID-19 pandemic but significantly lower than the reports during the peak of the pandemic [22]. This suggests that under regular working conditions (excluding major public health crises), work-family conflict is prevalent among emergency nurses, yet its intensity may be moderated by macro environmental pressure. In terms of demographic factors, our findings supported and partially extended the existing literature. First, male nurses had significantly higher conflict scores than female nurses, which was consistent with the study by Diao et al. [23]. Females are generally considered to bear more family responsibilities, but the unique high-pressure work environment of emergency departments, which often involves physical confrontation, may expose male nurses to a stronger conflict between the role expectation of “masculinity” and occupational reality, leading to more severe role imbalance and emotional exhaustion, and ultimately manifesting as higher work-family conflict. Second, nurses aged 30–40 years, married, with multiple children, and working more than 40 h per week had more prominent conflict. This was fully consistent with the predictions of the life course and role accumulation theory: nurses in this age group are in a critical period of career development and a peak period of family responsibilities, and multiple roles compete fiercely with limited time resources. Notably, working more than 40 h per week was a strong predictor of conflict, which directly confirmed that time-based conflict is the most fundamental dimension of work-family conflict and provided the most direct entry point for management interventions (e.g., flexible scheduling and working hour control).

### Correlations among organizational trust, affective rumination, problem-solving pondering and work-family conflict in emergency department nurses

This study revealed an opposite correlation pattern between organizational trust and the two types of rumination, which is the key to understanding how the organizational environment shapes individual cognitive strategies. Organizational trust was negatively correlated with affective rumination (*r*=-0.337) and positively correlated with problem-solving pondering (*r* = 0.437). This finding provided a more sufficient explanation for the study by Bakker et al. [24]: as a core work resource, high-level organizational trust can not only directly offset work demands but also guide individuals’ cognitive resources from passive and consumptive affective rumination to active and constructive problem-solving pondering. This cognitive “diversion” effect is the internal psychological mechanism underlying the positive impact of organizational trust. In addition, the relationships between affective rumination, problem-solving pondering and work-family conflict reconfirmed the “double-edged sword” characteristic of work rumination [13]. This study quantified the specific effects of this “double edge” in the emergency nurse group for the first time: affective rumination acts as an “accelerator” of resource depletion, while problem-solving pondering may serve as a “buffer” of resource construction under specific conditions. However, a potential contradiction that must be addressed is that excessive problem-solving pondering, or that occurring in non-working hours without the possibility of practical solution, may itself erode psychological boundaries. The negative correlation between organizational trust and work-family conflict in the model of this study may be attributed to the expectation of “problems being likely to be solved” provided by high organizational trust, thus making its positive aspects dominant. Future research needs to explore the boundary conditions under which such “constructive pondering” transforms into “intrusive worry”.

### Parallel mediating effects of work rumination between organizational trust and work-family conflict among emergency department nurses

This study revealed the detailed mechanism by which organizational trust affects work-family conflict through two parallel paths: affective rumination and problem-solving pondering, and fully interpreted the “double-edged sword” effect of work rumination. Among them, the affective rumination path (effect value = -0.088) reflected a typical process of resource depletion: low organizational trust is prone to induce repeated immersion in negative work emotions, which continuously consumes psychological resources and erodes the work-family boundary, thereby exacerbating conflict. The problem-solving pondering path (effect value = -0.131) exhibited more complex adaptive characteristics: a high-organizational-trust environment prompts nurses to engage in constructive thinking about work problems, which is essentially a process of resource acquisition and construction, enhancing the sense of work control and reducing stressors from the source, thus showing a significant adaptive role in this study. Nevertheless, the “double-edged sword” potential of problem-solving pondering must be understood dialectically—without effective boundary management, excessive problem-solving thinking may develop from cognitive infiltration to psychological detachment disorder, thereby potentially weakening its positive effects. Therefore, organizational trust not only directly buffers conflict but also protects nurses’ work-family balance through multiple paths by inhibiting maladaptive rumination, promoting adaptive rumination, and guiding it to remain within healthy boundaries. This suggests that management practices need to simultaneously optimize the organizational trust environment and carry out cognitive boundary management training to maximize the effectiveness of interventions.

## Limitations

This study had certain limitations. First, as a cross-sectional study, it could not infer the causal relationship between variables. Future research should adopt longitudinal or experimental designs to verify the causal directions in this model. Second, convenient sampling was used, and all samples were from Chongqing, which limited the generalizability of the research conclusions to emergency nurse groups in other regions and cultural backgrounds. Future multicenter randomized sampling studies can improve the generalizability. Third, there was a risk of common method bias: all data were collected through a one-time self-report by nurses, which may be affected by common method bias. Although statistical tests showed no serious problems, future research should include multi-source data to enhance validity. Fourth, there were uncontrolled potential confounding factors: the model did not include important covariates that may affect work-family conflict, such as personal resilience, family support, and socioeconomic status, which may affect the robustness of the model estimation.

## Conclusion

This study initially revealed a detailed path through which organizational trust affects work-family conflict among emergency department nurses: it exerts an indirect protective effect by inhibiting maladaptive affective rumination and promoting adaptive problem-solving pondering. Despite the aforementioned methodological limitations, the research results link organizational-level trust building with individual-level cognitive management, providing an integrated theoretical framework and specific targets for designing multi-level interventions to alleviate work-family conflict among emergency department nurses. Future intervention research can build on this to explore the effectiveness of comprehensive programs combining the construction of a trust environment with the training of cognitive boundary management skills.

## Data Availability

Due to privacy protection and ethical restrictions, the research data are not publicly available. For legitimate research purposes, interested parties may contact the corresponding author to inquire about the potential for data access.

## Abbreviations

ED: Emergency Department
CFA: Confirmatory Factor Analysis
RMSEA: Root Mean Square Error of Approximation
CFI: Comparative Fit Index
TLI: Tucker-Lewis Index
SRMR: Standardized Root Mean Square Residual
CR: Composite Reliability
AVE: Average Variance Extracted
WRS: Work Rumination Scale
WFCS: Work-Family Conflict Scale
SPSS: Statistical Package for the Social Sciences
VIF: Variance Inflation Factor
AMOS: Analysis of Moment Structures
SEM: Structural Equation Model
CI: Confidence Interval
WFC: Work-Family Conflict
IFI: Incremental Fit Index
